# First molecular identification and phylogenetic analysis of *Neospora caninum* in dogs from Sulaymaniyah province, Iraq

**DOI:** 10.2478/jvetres-2026-0001

**Published:** 2026-01-13

**Authors:** Aram Ahmad Mohammed, Taib Ahmed Hama Soor

**Affiliations:** Department of Microbiology, College of Veterinary Medicine, University of Sulaimani, Sulaymaniyah 46001, Iraq; Medical Laboratory Department, College of Health and Medical Technology, Sulaimani Polytechnic University, Sulaymaniyah 46001, Iraq

**Keywords:** abortigenic disease, canine parasite, *Nc5* gene, partial Sanger sequencing, phylogeny

## Abstract

**Introduction:**

*Neospora caninum* is a cosmopolitan intracellular coccidian parasite causing paralysis and neuromuscular problems in dogs, as well as abortion in cattle. This study investigated the molecular prevalence of neosporosis in Sulaymaniyah, Kurdistan, Iraq, and characterised the genetic diversity of the *N. caninum Nc5* gene in isolates from that region.

**Material and Methods:**

From July to December 2024, a study was conducted in Sulaymaniyah province in which 258 canine blood samples were collected and analysed for *N. caninum* infection. Conventional PCR and *Nc5* gene sequencing were used to detect the parasite and assess its genetic diversity.

**Results:**

The rate of infection with *N. caninum* was 3.87% and the disease was significantly (P < 0.05) higher in stray dogs (6.38%) than in kept dogs (0.85%), in dogs (6.45%) than in bitches (1.49%) and in adults (6.30%) than in juveniles (1.52%). Analysis of partial *Nc5* gene sequences from the protozoa resulted in the isolation and identification of 10 local isolates in the study region. Six of these isolates matched sequences previously deposited in GenBank, while four novel isolates, characterised by six new mutations, were identified for the first time in the research area. Phylogenetic analysis indicated a close relationship between *N. caninum* isolates from different countries.

**Conclusion:**

The current research provided a useful molecular dataset for *N. caninum* species, which is crucial for the assessment of phylogenetic associations and the molecular identification of these protozoans.

## Introduction

*Neospora caninum* is an obligate intracellular protozoan parasite within the Apicomplexa phylum (9). It is an organism with a two-host life cycle: dogs (or wild canids) are the definitive host and cattle (other ruminants) are the intermediate hosts (12). Although it can cause severe disease in dogs of all ages, infections are most frequently observed in puppies (26). The infection often presents as neuromuscular disorders, including encephalomyelitis and myositis, which can lead to paralysis and premature mortality in affected puppies (3).

Neosporosis is predominantly recognised as a cattle disease and is a significant contributor to substantial economic losses on cattle farms (14). Neosporosis is a globally distributed abortigenic disease (13), with reported cases in Europe, Canada, the USA, Australia, Costa Rica, South Africa and Japan (11). Infections in dogs have been documented in numerous countries, such as Brazil (22), Portugal (5), Turkey (35), Iran (21, 25) and Iraq (18, 24).

Since the clinical signs of *N. caninum* infection are not sufficient for establishing its diagnosis, serological tests have been used including immunofluorescence antibody tests, nucleic acid testing, Western blotting and ELISAs paired with molecular techniques to give accurate diagnoses in dogs (31, 35). The high level of sensitivity of PCR made it a viable alternative to morphological methods in the detection of *N. caninum* infection (27) and also useful in the detection of other parasites both in dogs and intermediate hosts.

This study aimed at the evaluation with a molecular technique of the prevalence of neosporosis in Sulaymaniyah, Kurdistan, Iraq and characterisation of the genetic diversity of the *N. caninum Nc5* gene in isolates from that region. To the best of our knowledge, this study is the first to report the molecular detection of *N. caninum* in the area’s dogs. It is an initial molecular investigation using PCR amplification and partial sequencing of the *Nc5* gene of *N. caninum* in this region and includes an assessment of associated risk factors in dogs for infection.

## Material and Methods

### Study region and sampling

Blood specimens were taken from 258 dogs in Sulaymaniyah province, Kurdistan Region, northeastern Iraq (35°04′–36°30′N, 44°50′–46°16′E) between July and December 2024. The sampled population comprised 124 dogs and 134 bitches which were 131 juveniles (12 months or younger) and 127 adults (over 12 months old), and included both stray (n = 141) and domestic (n = 117) animals. Risk factors for neosporosis considered in this study included lifestyle differences between stray and domestic dogs, age-related variations in immune response, and sex differences as hormonal and behavioural factors. Buffy coats were extracted from the blood samples and stored at -20°C until analysis for *N. caninum* infection.

### Molecular analysis

Genomic DNA was extracted from the buffy coat samples of all 258 dogs using FavorPrep blood genomic DNA extraction kits (Favorgen Biotech Corp, Pingtung City, Taiwan) according to the manufacturer’s protocols. Amplification of the *Nc5* gene in a PCR was performed using NP6 (5′-CAGTCAACCTACGTCTTCT-3′) and NP21 (5′-GTGCGTCCAATCCTGTAAC-3′) primers as described by Yamage *et al*. (34). The amplification was conducted using f-Pfu DNA polymerase (SBS Genetech Co., Beijing, China) applying the conditions described by Hariri *et al*. (21). Amplified products were visualised on 1.5% agarose gels stained with GelRed (Biotium, Fremont, CA, USA), revealing the expected 328 bp fragment.

Ten PCR amplicons were selected for sequencing. Fragments of the DNA were purified from agarose gels using the SiMax PCR Products/Agarose Gel Purification Kit (SBS Genetech, Beijing, China). Partial Sanger sequencing was performed using the upstream primer (Macrogen, Seoul, South Korea). The obtained sequences were edited and aligned using the ClustalW multiple sequence alignment algorithm. All sequences were subsequently deposited to GenBank.

Sequence similarity analysis was performed using BLAST to compare our *N. caninum Nc5* gene sequences with previously published sequences in GenBank. Phylogenetic analysis was conducted by aligning the coding sequences with reference *N. caninum* sequences (Table 1) using the maximum likelihood method. The phylogenetic tree was constructed using MEGA 11 software (32), with genetic distances calculated using Kimura’s two-parameter model. Tree topology reliability was evaluated through 1,000 bootstrap replicates.

**Table 1.** Global *Neospora caninum* GenBank sequences and accession numbers used for phylogenetic analysis and multiple sequence alignment

Protozoan species	GenBank accession No.	Geographical origin	Host organism	Citation
*N. caninum*	OP244818	Iraq	Sheep	Essa (17)
	MT955657		Cattle	Gharekhani *et al*. (19)
	MT955656	Iran		
	MT709298			
	MT709296		Dog	
	MT346029	Italy	Red fox	Zanet *et al*. (36)
	KP715569	Italy	Deer	Zanet *et al*., 2015, unpublished
	LN714476	UK	-	Ramaprasad *et al*. (29)
	KF649845	USA	Wolf	Dubey *et al*. (10)

### Statistical analysis

A chi-squared (χ^2^) test was used to determine significance at P-value < 0.05.

## Results

### Molecular prevalence of *N. caninum*

Amplification of the *Nc5* gene in a PCR produced a 328-bp fragment, although sequencing analysis revealed a 305-nucleotide sequence. Among the 258 dog blood samples tested, *N. caninum* DNA was detected in 10, representing a prevalence of 3.87%. Risk factors associated with neosporosis were investigated by analysing infection rates based on lifestyle, age and sex (Table 2).

**Table 2.** Association of risk factors with *N. caninum* infection in dogs from Sulaymaniyah Province, Iraq

		Number of dogs examined	Number (%) of dogs infected	*X*^2^ (P-value)
	Overall	258	10 (3.87)	
Lifestyle	Stray dogs	141	9 (6.38)	0.022 (<0.05)
	Domestic dogs	117	1 (0.85)	
Sex	Male	124	8 (6.45)	0.039 (<0.05)
	Female	134	2 (1.49)	
Age	Young (≤12 months)	131	2 (1.52)	0.047 (<0.05)
	Adult (>12 months)	127	8 (6.30)	

The study revealed that these were significant risk factors for *N. caninum* infection in dogs. Specifically, stray dogs exhibited a significantly higher infection rate (6.38%) than domestic dogs (0.85%; P < 0.05). The study also found a significantly higher *N. caninum* infection rate in dogs (6.45%) than in bitches (1.49%; P < 0.05). Additionally, adults showed a significantly greater infection rate (6.30%) than juveniles (1.52%; P < 0.05).

### Sequence analysis of *N. caninum*

**Fig. 1.**
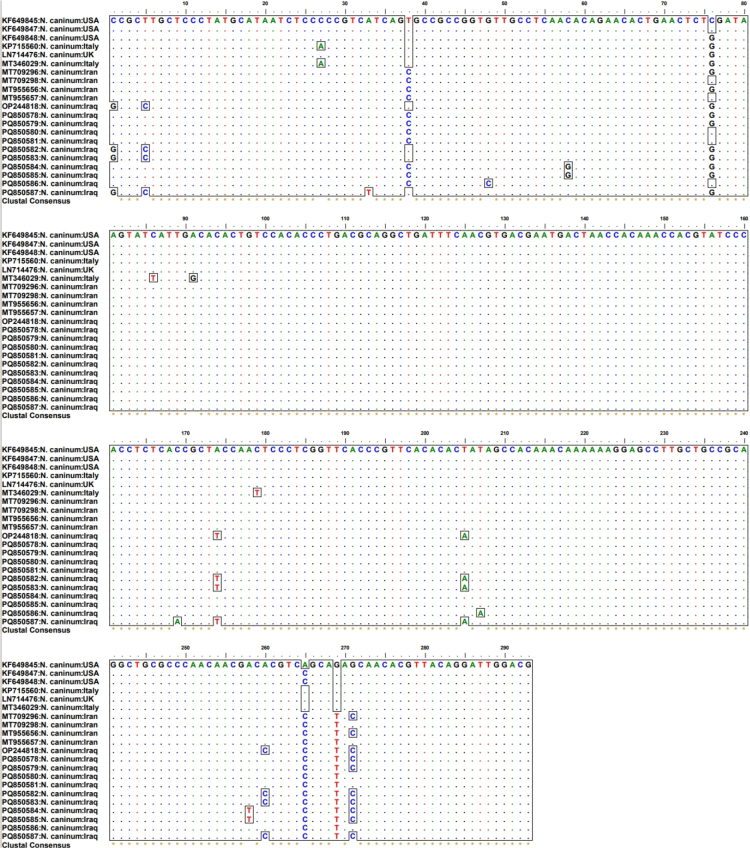
Multiple sequence alignment of *Neospora caninum* isolates from dogs in Sulaymaniyah, Iraq, alongside other GenBank isolates. The alignment was conducted using the ClustalW algorithm on partial *Nc5* gene sequences. The nucleotide sequences from this study are assigned accession Nos: PQ850578–PQ850587

**Fig. 2.**
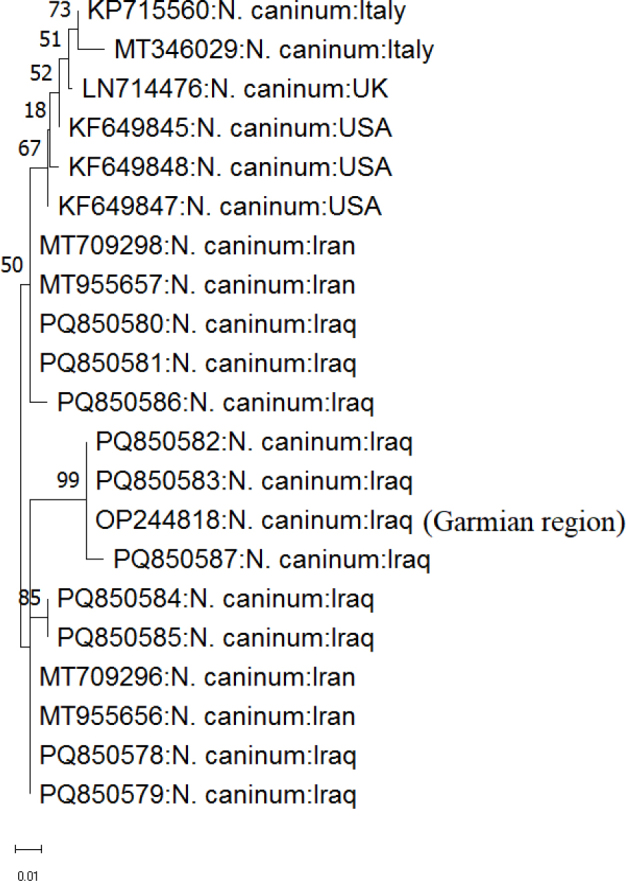
Phylogenetic tree of *Neospora caninum* isolates from dogs in Sulaymaniyah, Iraq, constructed using the maximum-likelihood method based on *Nc5* gene sequences. The accession Nos: PQ850578–PQ850587 represent the sequences analysed in this study. The scale bar indicates a 1% nucleotide difference

Sequence editing and trimming yielded a 293-bp PCR product. Sequence analysis and alignment revealed six distinct *N. caninum* isolates with variations, including several mutations (Fig. 1). The *Nc5* gene in this study comprised ten isolates which were assigned GenBank accession Nos PQ850578–PQ850587. The isolates from this study fell into two groups. Four isolates (PQ850584–PQ850587), found only in the research area, presented six novel mutations: at positions 58 (C > G) and 258 (A > T) in PQ850584 and PQ850585, positions 48 (G > C) and 207 (T > A) in PQ850586 and positions 33 (A > T) and 169 (C > A) in PQ850587. The other six isolates (PQ850578–PQ850583) had 97.38%–100% identity with the previously identified sequences with accession numbers MT 709296, MT709298, MT955656 and MT955657 from Iran, and OP244818 from Iraq (Fig. 1). Analysis by BLAST demonstrated that all sequences exhibited high similarity (95.41% to 100%) to *N. caninum* sequences available in GenBank, the output of closest sequences being examples from Iran, Iraq, Italy, the UK and the USA (Fig. 1). Phylogenetic analysis of the *Nc5* sequences showed that the *N. caninum* isolates sequenced in the present research were distributed across different clades, reflecting genetic diversity within the country (Fig. 2).

## Discussion

Over the past three decades, *N. caninum* has been extensively researched as a significant veterinary pathogen (8). However, limited data is available on its occurrence in dogs and foxes, which are the parasite’s definitive hosts. Dogs, in particular, play a critical role in the epidemiology of neosporosis as the primary definitive hosts of *N. caninum* (31).

In this study, the overall molecular detection rate of *N. caninum* in dog blood specimens was 3.87% (10/258), which is similar to what a study recorded in Turkey (3.80%) (15). This rate is lower than those reported in Baghdad, Iraq (10%) (18); Tehran, Iran (35%) (28); Isfahan, Iran (24.40%) (25); and Botucato, Brazil (42.90%) (22). However, it is somewhat higher than the rates documented in the Al-Muthana province of Iraq (1.60%) (24) and the Van province of Turkey (2%) (35). Variations in molecular prevalence rates could be attributed to several associated risk factors, such as sex, breed, age, coinfections, intermediate host involvement, diet and climatic conditions. These elements can significantly influence transmission dynamics, such as the sporulation and viability of oocysts (4, 7, 31).

We also studied the effect of risk factors including life style, sex and age. Stray dogs had a significantly higher infection rate than domestic dogs (6.38% *versus* 0.85%, P < 0.05). This result agrees with the findings of Wang *et al*. (33), and Fanokh and Al-Rubaie (18). These notable findings may be attributed to consumption of raw meat containing tissue cysts, welfare conditions and living environment (33). Neosporosis was significantly higher in dogs than in bitches (6.45% *versus* 1.49%, P < 0.05). The results are in agreement with the earlier observations of Wang *et al*. (33), Adhami *et al*. (1) and Fanokh and Al-Rubaie (18). In contrast, Al-Amery *et al*. (2) in Iraq reported that the infection rate in female buffaloes was higher than that in male ones; also, our data are in disagreement with those of Gozdzik *et al*. (20) in Poland and Dwinata *et al*. (16) in Indonesia. The incidence rate of neosporosis was significantly higher in adults (6.30%) than in juveniles (1.52%; P < 0.05). This finding corroborates the results of the previous studies showing that the rate of neosporosis increased with dog age (18, 20, 33). This is plausibly due to transmission dynamics, immune response and the symptomatic nature of the diseases.

Genotyping with PCRs has become indispensable for genetic analysis because it requires minimal DNA but makes strain identification, tracking of genotype frequencies and the determination of alleles possible. These benefits have established investigation of genetic markers as the preferred approach for detecting genomic variations in eukaryotic organisms (23). Regidor-Cerrillo *et al*. (30) were the first to report the presence of repetitive sequences within the *N. caninum* genome, and further studies by Calarco *et al*. (6) have identified more than 100 distinct *N. caninum* strains. Molecularly, this study analysed the genetic diversity of *N. caninum* based on *Nc5* gene nucleotide sequences isolated from the blood of Iraqi dogs. The PCR analysis successfully amplified a 328-bp fragment of the target *N. caninum* gene in dog blood samples. and exposed its sequence to show genome arrangements typical of *N. caninum* sequences in GenBank. Sanger sequencing of the *Nc5* gene in our samples was used to confirm the PCR results; and it was substantiated that all the positive samples tested were *N. caninum*.

Next, we used multiple sequence alignment and a phylogenetic tree to look for sequence variations and the presence of single nucleotide polymorphisms. This study identified novel *N. caninum* isolates with unique mutations not previously reported from anywhere in the world. These isolates exhibited the greatest similarity to sequences from Iraq, Iran, Italy, the UK and the USA. These results demonstrate that the samples from different geographic areas may vary genetically. Phylogenetic analysis revealed intraspecific variations among all canine-hosted sequences obtained in this study. This contrasts with the findings of Fanokh and Al-Rubaie (18), who reported a lack of variation in *N. caninum* sequences isolated from Iraqi dogs.

The molecular alignment and phylogenetic tree analysis performed in this study indicate a strong relationship between Iraqi isolates and Iranian isolates. This finding likely reflects the long shared border and frequent trade between the countries, including the import of sheep and animal products, and carnivore movement between Iraq and Iran (17). The findings showed that the *N. caninum* isolates obtained in this research and from GenBank varied genetically, and are a foundation for further research to accrue regional data against which to validate the explanation of Al-Qassab *et al*. (3) of clades by geographical distribution, host range, and the parasite’s capacity for sexual reproduction.

## Conclusion

The outcomes of this research demonstrate that *N. caninum* is present among dogs in Sulaymaniyah province, Iraq. These infected dogs are likely to be a source of *N. caninum* transmission to cattle, potentially leading to bovine abortions. This research is the first to document molecularly *N. caninum* infection in dogs from this province and gives useful data on *N. caninum* in dogs in Iraq using barcode *Nc5* genomic sequences. Our results showed a close association between *N. caninum* from Iraq and *N. caninum* from different countries, although limited sequences were included in the dataset. The findings of the research show the usefulness of the *Nc5* gene as a diagnostic molecular marker for identifying and characterising *N. caninum* species. Further research is needed to better understand the epidemiology of neosporosis in this region and develop effective *N. caninum* infection prevention strategies.

## References

[1] Adhami G., Dalimi A., Hoghooghi-Rad N., Fakour Sh. (2020). Molecular and serological study of *Neospora caninum* infection among dogs and foxes in Sanandaj, Kurdistan Province, Iran. Arch Razi.

[2] Al-Amery A.M., Faraj A.A., Faleh I.B. (2016). Seroprevalence and histopathological study of neosporosis in water buffaloes (*Bubalus bubalis*) in Baghdad city, Iraq. J Anim Health Prod.

[3] Al-Qassab S.E., Reichel M.P., Ellis J.T. (2010). On the biological and genetic diversity in *Neospora caninum*. Diversity.

[4] Anvari D., Saberi R., Sharif M., Sarvi S., Hosseini S.A., Moosazadeh M., Daryani A. (2020). Seroprevalence of *Neospora caninum* infection in dog population worldwide: A systematic review and meta-analysis. Acta Parasitol.

[5] Basso W., Herrmann D.C., Conraths F.J., Pantchev N., Globokar M.V., Schares G. (2009). First isolation of *Neospora caninum* from the faeces of a dog from Portugal. Vet Parasitol.

[6] Calarco L., Barratt J., Ellis J. (2018). Genome wide identification of mutational hotspots in the apicomplexan parasite *Neospora caninum* and the implications for virulence. Genome Biol Evol.

[7] Collantes-Fernández E., Gómez-Bautista M., Miró G., Álvarez-García G., Pereira-Bueno J., Frisuelos C., Ortega-Mora L.M. (2008). Seroprevalence and risk factors associated with *Neospora caninum* infection in different dog populations in Spain. Vet Parasitol.

[8] Donahoe S.L., Lindsay S.A., Krockenberger M., Phalen D., Šlapeta J. (2015). A review of neosporosis and pathologic findings of *Neospora caninum* infection in wildlife. Int J Parasitol Parasites Wildl.

[9] Dubey J.P., Hemphill A., Calero-Bernal R., Schares G. (2017). Neosporosis in animals.

[10] Dubey J.P., Jenkins M.C., Ferreira L.R., Choudhary S., Verma S.K., Kwok O.C.H., Carstensen M. (2014). Isolation of viable *Neospora caninum* from brains of wild gray wolves (*Canis lupus*). Vet Parasitol.

[11] Dubey J.P., Lindsay D.S. (1996). A review of *Neospora caninum* and neosporosis. Vet Parasitol.

[12] Dubey J.P., Schares G. (2006). Diagnosis of bovine neosporosis. Vet Parasitol.

[13] Dubey J.P., Schares G. (2011). Neosporosis in animals – the last five years. Vet Parasitol.

[14] Dubey J.P., Schares G., Ortega-Mora L.M. (2007). Epidemiology and control of neosporosis and *Neospora caninum*. Clin Microbiol Rev.

[15] Düzlü Ö., İnci A., Yildirim A., Önder Z., Çiloğlu A. (2014). Investigation of *Neospora caninum* and *Toxoplasma gondii* tachyzoites in peripheral blood samples of dogs by TaqMan probe based Real Time PCR. Ankara Univ Vet Fak Derg.

[16] Dwinata M., Oka I.B., Agustina K.K., Damriyasa M. (2018). Seroprevalence *of Neospora caninum* in local Bali dog. Vet World.

[17] Essa H. (2023). Molecular identification of *Neospora caninum* in aborted sheep in Garmian Region/Kurdistan of Iraq. Biochem Mol Biol J.

[18] Fanokh M.A., Al-Rubaie H.M.A. (2022). *Neospora caninum* detection by nested PCR in domestic and stray dogs in Baghdad City. Int J Health Sci.

[19] Gharekhani J., Yakhchali M., Heidari R. (2022). Molecular detection and phylogenetic analysis of *Neospora caninum* in various hosts from Iran. Comp Immunol Microbiol Infect Dis.

[20] Gozdźik K., Wrzesien R., Wielgosz-Ostolska A., Bien J., Kozak-Ljunggren M., Cabaj W. (2011). Prevalence of antibodies against *Neospora caninum* in dogs from urban areas in Central Poland. Parasitol Res.

[21] Hariri M., Arefkhah N., Ghorbani F., Namavari M., Omidian M., Sarkari B. (2021). Molecular and serological evaluation *of Neospora caninum* infection in dogs from a rural setting in Fars province, southern Iran. Iran J Parasitol.

[22] Langoni H., Matteucci G., Medici B., Camossi L.G., Richini-Pereira V.B., Silva R.C. da (2012). Detection and molecular analysis of *Toxoplasma gondii* and *Neospora caninum* from dogs with neurological disorders. Rev Soc Bras Med Trop.

[23] MacLeod A., Melville S.E. (2004). Methods in Molecular Biology, Vol 270, Parasite Genomics Protocols.

[24] Mallah M.O., Dawood K.A., Alrodhan M.A. (2012). First isolation of *Neospora caninum* in dogs in Al-Muthana Province, Iraq. AL-Muthanna J Pure Sci.

[25] Motamedi A., Keihani P., Momtaz H. (2020). Molecular detection of *Neospora caninum* in infected dogs of Isfahan, Iran. J Zoon Dis.

[26] Nishimura M., Tanaka S., Ihara F., Muroi Y., Yamagishi J., Furuoka H., Suzuki Y., Nishikawa Y. (2015). Transcriptome and histopathological changes in mouse brain infected with *Neospora caninum*. Sci Rep.

[27] Orlandi P.A., Lampel K.A. (2000). Extraction-free filter-based template preparation for rapid and sensitive PCR detection of pathogenic parasitic protozoa. J Clin Microbiol.

[28] Pouramini A., Jamshidi Sh., Shayan P., Ebrahimzadeh E., Namavari M., Shirian S. (2016). Molecular and serological detection of *Neospora caninum* in multiple tissues and CSF in asymptomatic infected stray dogs in Tehran, Iran. Iran J Vet Med.

[29] Ramaprasad A., Mourier T., Naeem R., Malas T.B., Moussa E., Panigrahi A., Pain A. (2015). Comprehensive evaluation of *Toxoplasma gondii* VEG and *Neospora caninum* LIV genomes with tachyzoite stage transcriptome and proteome defines novel transcript features. PLOS One.

[30] Regidor-Cerrillo J., Pedraza-Díaz S., Gómez-Bautista M., Ortega-Mora L.M. (2006). Multilocus microsatellite analysis reveals extensive genetic diversity in *Neospora caninum*. J Parasitol.

[31] Reichel M.P., Ellis J.T., Dubey J.P. (2007). Neosporosis and hammondiosis in dogs. J Small Anim Pract.

[32] Tamura K., Stecher G., Kumar S. (2021). MEGA11: molecular evolutionary genetics analysis version 11. Mol Biol Evol.

[33] Wang S., Yao Z., Zhang N., Wang D., Ma J., Liu S., Zheng B., Zhang B., Liu K., Zhang H. (2016). Serological study of *Neospora caninum* infection in dogs in central China. Parasite.

[34] Yamage M., Flechtner O., Gottstein B. (1996). *Neospora caninum*: specific oligonucleotide primers for the detection of brain “cyst” DNA of experimentally infected nude mice by the polymerase chain reaction (PCR). J Parasitol.

[35] Yilmaz A.B., Göz Y., Kilinç Ö.O., Denizhan V. (2020). Molecular detection of *Neospora caninum* from naturally infected dogs in Van province, east Turkey. Ind J Anim Res.

[36] Zanet S., Poncina M., Ferroglio E. (2023). Congenital transmission of *Neospora caninum* in wild ungulates and foxes. Front Vet Sci.

